# When the Underdog Positioning Backfires! The Effects of Ethical Transgressions on Attitudes Toward Underdog Brands

**DOI:** 10.3389/fpsyg.2020.01988

**Published:** 2020-09-04

**Authors:** Yaeri Kim, Kiwan Park

**Affiliations:** ^1^Department of Marketing, College of Business Administration, Sejong University, Seoul, South Korea; ^2^Department of Digital Marketing, School of Management, Sejong Cyber University, Seoul, South Korea; ^3^Department of Marketing, College of Business Administration, Seoul National University, Seoul, South Korea

**Keywords:** underdog backfiring, consumer brand identification, symbolic brand, ethical transgression, perceived betrayal, consumer brand attitude change

## Abstract

This research investigates the novel link between consumers’ support for underdog brands and their ethical expectations of such brands and finds that the underdog brand positioning may not always be beneficial. Rather, we argue that the identification-based supporting motivation for underdog brands may backfire when the accompanying specific moral expectation is not satisfied. Study 1 demonstrates that the underdog brand falls into an ethical trap in which consumers judge the brand more harshly when ethical transgressions are committed. In Study 2, the psychological underlying mechanism for this ethical underdog trap effect is proved to be perceived betrayal. In Study 3, a boundary condition, community-related (vs. autonomy-related) transgressions, is explored. In Study 4, the three types of transgressions (autonomy, community, and functional) and the mediating effects of perceived betrayal are tested in integrated research design. Finally, theoretical and managerial implications are discussed, followed by conclusions.

## Introduction

Underdog brand positioning and its positive effects have been investigated in many studies that have confirmed consumers’ support motivations for underdog brands (McGinnis and Gentry, 2009; Paharia et al., 2011; Kao, 2015; McGinnis et al., 2017; Goldschmied et al., 2018). However, none of the previous studies have mentioned potential risks involved with the underdog positioning, particularly in the moral domains. Kim et al. (2019) first announced and demonstrated the existence of the underdog trap by warning about the side effects of underdog positioning, but their focus was not on ethical transgressions. Although Kirmani et al. (2017) touched on moral issues, they focused on describing the underdog positioning as a breakthrough to overcome deficits in competence, thereby attenuating consumers’ tendency to seek highly competent brands rather than moral brands. The current research contributes to the literature by suggesting a novel linkage between the consumers’ supporting motivations toward the underdog brand and the ramifications of ethical expectations and the effect of committing transgressions. We argue that the identification-based supporting motivation behind the underdog orientation can positively affect the underdog brand but may work as a trap when the accompanying moral expectations are not satisfied.

Consumers tend to feel like underdogs in everyday life. Their disadvantaged experiences in society become a driving force in their positive attitudes toward underdogs (McGinnis and Gentry, 2009; Paharia et al., 2011; Kao, 2015). According to the literature on interpersonal reactivity, accurate perspective, judgment, and response are possible when individuals experience a similar situation; thus, greater empathy is anticipated from those in identical situations (Bernstein and Davis, 1982; Davis, 1983; Snodgrass, 1985; Piff et al., 2010). Applying these principles to the current research, people view underdog brands as representative of themselves and thus believe that these brands better understand them than others, such as top-dog brands, that have high resources and privilege.

However, if consumers observe underdog brands’ bad behavior in the moral domain, the supporting motivation that makes the underdog brand lovable can hurt it, creating severe brand damage through a boomerang effect. Consumers may feel betrayed, and this negative emotion can backfire on the underdog brand; that is, “the higher you are, the harder you fall” (Brockner et al., 1992, p. 241). We consider this negative attitude change toward the underdog brand to be *the ethical underdog trap*. We argue that the asymmetrically higher moral standards for the underdog brand is particularly pronounced for autonomy-related ethical transgressions (vs. community-related ethical transgressions or functional transgressions), as these are more closely related to the supporting motivation behind the underdog orientation (Shweder et al., 1997; Rozin et al., 1999; Grappi et al., 2013). Autonomy rights are basic human rights respected by empathic concerns. Given consumers’ high identification with underdog brands, they may require the same moral standards and perspective-taking from these brands as they do for people (Waytz et al., 2010; Kwak et al., 2017; MacInnis and Folkes, 2017; Kouchaki et al., 2018; Lin and Huang, 2018; Teresi et al., 2019). On the other hand, consumers do not necessarily hold top-dog brands to such moral standards or expect them to make efforts to be perspective of others’ autonomy rights. In this research, we explore a boundary condition in which the ethical underdog trap is (not) observed and whether this effect can be explained by perceived betrayal.

## Literature Review and Hypotheses

### Types of Transgressions and Alignment With Brand Positioning

Previous research suggests that transgressions have diverged into two major streams. One is performance-related transgressions and the other is value-related transgressions (Pullig et al., 2006; Dutta and Pullig, 2011). Performance-related transgressions are directly related to the core function of the product or the basic service need (Dawar and Pillutla, 2000; Pullig et al., 2006; Roehm and Brady, 2007). This type of transgression relates to a particular attribute that solves consumers’ specific consumption problems by providing functional benefits (Dawar and Pillutla, 2000). Examples of performance-related transgressions include the failure of an automobile part or the detection of lead in toys. In contrast, value-related transgressions do not directly encompass the product or its functional ability, but rather involve problems surrounding values encapsulated by the brand, which affect the brand’s symbolic benefits that allow for the reflection of self-image (Pullig et al., 2006).

According to Roloff et al. (2001), committing transgressions – the violation of rules governing a relationship – allows people to learn about the qualities of others involved in the relationship and affords them the opportunity to investigate a wide range of unpredicted and inappropriate behaviors across diverse relationship types (Metts, 1994). The consumer–brand relationship is no exception, but despite its importance, the empirical investigations of the consumer–brand relationship regarding transgressions are largely focused only on the service domain (Smith et al., 1999; Hedrick et al., 2007) and consumers’ post-transgression reactions (Sinha and Lu, 2016). Thus, understanding the influence of various types of transgressions (mis)aligned with brand positioning in the consumer–brand relationship, as well as the underlying mechanism explaining the influence (Aaker et al., 2004), is limited.

The literature suggests that brand positioning aligned with the transgression will incur a negative impact on brand attitude. For instance, extending the information matching-and-mismatching principle (Anderson, 1981; Edwards, 1990; Petty and Wegener, 1998; Fabrigar and Petty, 1999) to brand positioning and negative brand publicity, Pham and Muthukrishnan (2002) presented a search-and-alignment model. According to this model, when challenging information is aligned with existing pro-attitude information, it is more likely to engender detailed elaboration about the challenge (Fabrigar and Petty, 1999) and receive biased weight in judgment (Muthukrishnan et al., 1999). Pham and Muthukrishnan (2002) further concluded that when the brand positioning (performance vs. value) and subsequent challenge information (performance vs. value challenge) are aligned, consumers perceive matching challenges as more diagnostic and express their intentions with a greater change to their brand attitude, as opposed to cases in which positioning and challenge are mismatched or unaligned. In the moral domain, how consumers combine preexisting (un)ethical brand perception and subsequent inconsistent valenced information has been investigated through the lens of person–perception formation (Berens et al., 2005; Brunk and de Boer, 2018).

In developing our prediction about the role of brand biography, it is important to understand the connotations that the underdog vs. top-dog positioning project to consumers. The underdog brand positioning does not focus on specific attributes, but rather stresses the underdog’s humble background and noble, passionate, determined struggles. This positioning delivers symbolic benefits (Paharia et al., 2011; McGinnis et al., 2017), and the related positive effect is explained by the identity mechanism (McGinnis and Gentry, 2009; Paharia et al., 2011; Kao, 2015) grounded in the self–brand connection principle (Fournier, 1998; Aaker et al., 2004). Consumers support the underdog because its story reflects the underdog aspects of the consumers’ own lives. Feeling empathy through the shared experience of the disadvantaged position is not difficult, and this drives the consumers’ positive attitude toward the underdog (McGinnis et al., 2017). Thus, as discussed earlier, when value-related ethical transgressions (matching information) are committed, the symbolic positioning of the underdog brand may face critical judgments from consumers (Gaustad et al., 2019). The impact of functional transgressions (mismatching information) on the underdog brand positioning may be attenuated.

Top-dog brand characteristics, such as few external disadvantages and low passion and determination (Paharia et al., 2011), have been reflected in well-known brands such as Wal-Mart. In contrast to underdog brands, the supporting motivation for top-dog brands may be based on the “basking in reflected glory” effect (Cialdini et al., 1976; Cialdini and de Nicholas, 1989). For instance, students were more likely to prefer a university’s logo that presented a victory rather than a loss (Cialdini et al., 1976), and study participants were reluctantly associated with losing sports teams (Cialdini and Richardson, 1980). Furthermore, although we may want to be in the top-dog position with abundant resources and the privileged ability to defeat competitors, life experience does not often allow us that luxury. Thus, identifying and sympathizing with the position of the top dog is not as easy as it would be with the underdog. Rather, we vaguely expect quality products or services to be delivered from the top dog because they possess sufficient resources. Thus, the top-dog positioning cannot be considered to deliver symbolic benefits of self-expression or reflect one’s identity, but instead deliver functional benefits (Bhat and Reddy, 1998).

### Do Ethical Transgressions Always Damage Underdog Brands?

It may be argued that value-related ethical transgressions do not always negatively affect the underdog brand. Two types of ethical transgressions are distinguished to address the problem. One type is committed when corporate behaviors violate autonomy or individual freedom rights, which we term as autonomy-related ethical transgressions. The other type occurs when a corporation deliberately damages a certain community by violating rules or standards expected in society, which we term as community-related ethical transgressions (Shweder et al., 1997; Rozin et al., 1999; Grappi et al., 2013). We contend that severely negative attitude changes are induced under autonomy-related (vs. community-related) ethical transgressions. This reasoning is based on consumers’ basic supporting motivation toward the underdog brand (McGinnis and Gentry, 2009; Paharia et al., 2011; Kao, 2015). Identification with the underdog is the very mechanism that may explain the backfiring effect on the underdog positioning in the case of autonomy-related ethical transgressions.

It is impossible to control everything in our lives. We are forced to stand on our own, but we often fall under the pressure of countless difficulties. We often feel like underdogs rather than top dogs. Therefore, it is easy to sympathize with underdog brands that have less resources and lower positions yet remain passionate and determined. The supporting motivation toward the underdog does not lie in pursuing practical or utilitarian brand benefits (Paharia et al., 2011). Rather, the supporting motivation relates to symbolic benefits that give us the feeling of being in the same boat. Hence, underdog brands that share similar difficult experiences as identified by consumers are perceived through the same lens that people use to view human beings. Thus, it may not be acceptable for *underdog* brands to infringe on individuals’ autonomy rights, and equal or fair opportunity rights, as people in the underdog position are expected not to do so.

The literature on interpersonal reactivity also provides a foundation to support our prediction. According to Snodgrass (1985), people tend to increase sensitivity to others when they are in a subordinate role or have feminine intuition. Similarly, lower-class individuals tend to have greater compassion when it comes to the needs of others and are more likely to act in a pro-social fashion (Piff et al., 2010). That is, people in lower positions are more likely to empathize strongly with others based on perspective-taking and to transpose their views to other imaginary characters or create empathic concern (Bernstein and Davis, 1982). Thus, a high interpersonal sensitivity is expected even for underdog brands, which leads to the expectation of greater empathic concern. This expectation should be well-represented in autonomy-related ethical problems where basic human rights are to be respected. Defending the autonomy of others does not necessarily require a high power or privileged status, whereas influencing the rights of others may need force. Just a minimum level of good intentional effort can acknowledge others’ individual freedoms.

In contrast, although community-related ethical transgressions are subsumed in value-related transgressions, the degree of negative emotion toward the underdog is not expected to exceed the threshold to negative attitude change, and its effect should be less than in the case of autonomy-related ethical transgressions (Rozin et al., 1999; Grappi et al., 2013). Rather, top-dog brands are likely to be damaged by committing community-related transgressions. It is generally believed that in the decision-making process, people often intentionally control others by developing policies or rules (Van den Bos and Lind, 2002; Van Prooijen and Lam, 2007). Likewise, people who have higher social status have a greater chance of exploiting others (Van Vugt et al., 2008; Boehm and Boehm, 2009). Thus, top-dog brands that have a huge influence on society should abide by community norms as they are influential members of society. Thus, the top-dog position seems more vulnerable to the consequences of violating community norms.

### Perceived Betrayal as the Underlying Mechanism

We put forth our assumption that the underdog will be judged harshly for committing autonomy-related transgressions by incorporating the underlying mechanism of perceived betrayal. In this research, perceived betrayal is defined as the consumer’s belief that a corporation has deliberately violated what are generally accepted as norms in the consumer–brand relationship (Elangovan and Shapiro, 1998; Koehler and Gershoff, 2003; Ward and Ostrom, 2006). Research on betrayal has examined close human relationships (Finkel et al., 2002) and the relationships between employee and employer (Elangovan and Shapiro, 1998). Perceived betrayal is referred to as the mechanism that accounts for the online consumer’s protest (Ward and Ostrom, 2006) and retaliation (Grégoire and Fisher, 2008). The findings from these studies consistently argue that the act of betrayal is hard to forgive or forget. As alluded to in its definition, perceived betrayal occurs in an established relationship and provides a clue to the “love-becomes-hate” effect (Grégoire and Fisher, 2008, p. 258). The love-becomes-hate effect is interesting in that the quality of the relationship may not be able to alleviate offensive feelings, but may rather amplify them. Thus, consumers who feel a strong connection with a brand feel more upset when they believe they are being treated poorly.

**FIGURE 1:**
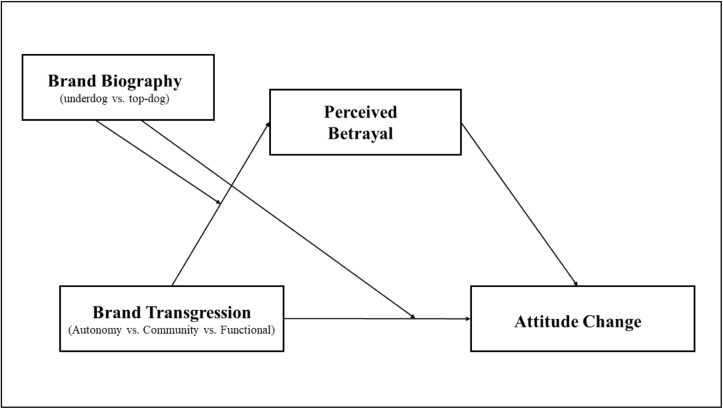
Conceptual framework.

A similar negative effect may occur for strongly identified relationships. We feel bitter when we are betrayed by a friend. Findings in social psychology also support our assumption. Individuals are more likely to feel extremely offended when they are criticized by a group they are strongly attached to (Moreland and McMinn, 1999) or by a transgressor with whom they perceive they have a quality relationship (McCullough et al., 1998). Further, for highly identified firms, negative information drives consumers to feel betrayed and to react strongly in a more permanent way (Bhattacharya and Sen, 2003). Therefore, in the highly identified relationship with underdog brands, the sense of betrayal from consumers may be amplified when the brands commit autonomy-related ethical transgressions, which in turn leads to a negative effect on brand attitude. The violation of ethics related to autonomy creates a greater sense of betrayal among consumers, thereby undermining consumer motivation to support underdog brands. The classic literature on social exchange theory (Blau, 1962) makes our assumption plausible. As in strong relationships, consumers may feel that the brands owe them and expect to be treated accordingly. Thus, we posit the following hypotheses (Figure 1):

H1 Consumers will change their attitudes more negatively when underdog brands commit ethical (vs. functional) transgressions. Transgression type will not affect attitude change toward top-dog brands.

H2 Perceived betrayal mediates the moderating effect of transgression type on consumers’ negative attitude change.

H3 Consumers will change their attitudes more negatively when underdog brands commit autonomy-related (vs. community-related) ethical transgressions. Ethical transgression type will not affect attitude change toward top-dog brands.

### Overview of Studies

We tested these hypotheses in four experimental studies. Before conducting the main studies, we did a pilot test to examine whether consumers had different moral expectations about underdog vs. top-dog brands. In Study 1, we examined whether the underdog positioning would face harsh judgment from consumers regarding ethical (vs. functional) transgressions. Study 2 was designed to uncover the underlying mechanism driving the negative attitude change, as observed in Study 1. In Study 3, a boundary condition for the proposed effect of the ethical underdog trap was explored. We predicted that not all ethical transgressions would serve as disadvantages to the underdog positioning. We tested whether the proposed effect would be present only in autonomy-related (vs. community-related) ethical transgressions. The purpose of Study 4 was to replicate the proposed effect in an integrated research design that included all the transgression types of Studies 1–3 and also to replicate the previous results. The overview of the studies is described in Table 1.

**TABLE 1:** Overview of the studies.

Studies	Study design
**Study 1**: The ethical underdog trap	2 (transgression type: ethical vs. functional) × 2 (brand biography: underdog vs. top dog) between-participants design
**Study 2**: The mediating role of perceived betrayal	
**Study 3**: A boundary condition of the ethical underdog trap	2 (ethical transgression type: autonomy vs. community) × 2 (brand biography: underdog vs. top dog) between-participants design
**Study 4**: The ethical underdog trap in an integrated design	3 (transgression type: autonomy vs. community vs. functional) × 2 (brand biography: underdog vs. top dog) between-participants design

## Pilot Test

We posted the brand perception question to online panel members consisting of national university students. Sixty students participated (*M*_*age*_ = 25.48, *SD* = 2.97; 34 females). Participants read one of the two brand biography scenarios: one describing the underdog and the other the top dog. They next evaluated their perceptions toward one of the brands on a 7-point scale: “How do you perceive the brand in general?” (1 = not at all moral, 7 = highly moral). The results found overall moral expectation toward the underdog and the top dog did not differ [*M*_*underdog*_ = 5.00 vs. *M*_*top–dog*_ = 4.50; *F*(1, 58) = 0.458, *p* = 0.501].

However, the expectation toward each brand was different for autonomy-related moral values. Participants did believe that the underdog brand should be more responsible for autonomy-related ethical issues (four items on a 7-point scale; 1 = strongly disagree, 7 = strongly agree), such as providing equal opportunities for social members [*M*_*underdog*_ = 5.03 vs. *M*_*top–dog*_ = 3.25; *F*(1, 58) = 29.80, *p* < 0.001], protecting against human indignity [*M*_*underdog*_ = 4.41 vs. *M*_*top–dog*_ = 3.46; *F*(1, 58) = 8.866, *p* = 0.004], supporting fair competition [*M*_*underdog*_ = 5.59 vs. *M*_*top–dog*_ = 3.04; *F*(1, 58) = 73.909, *p* < 0.001], and defending individual autonomy rights [*M*_*underdog*_ = 4.13 vs. *M*_*top–dog*_ = 3.36; *F*(1, 58) = 5.215, *p* = 0.026].

## Study 1: The Ethical Underdog Trap

Study 1 was designed to confirm that in the ethical underdog trap effect, the underdog biography negatively affects firms when the underdog brand commits ethical transgressions. Consistent with H1, we predicted that people would judge underdog brands more harshly for committing ethical transgressions vs. functional transgressions.

### Materials and Methods

#### Participants, Design, and Procedure

One hundred two undergraduates from a major university in South Korea (*M*_*age*_ = 23.95, *SD* = 3.33; 57 females) participated in an online experiment using Qualtrics software. The participants were randomly assigned to one of four conditions in a 2 (transgression type: ethical vs. functional) × 2 (brand biography: underdog vs. top dog) between-participants design. First, participants were assigned to one of two brand biography conditions (Paharia et al., 2011, Supplementary Appendix A) in which a hypothetical “Company A” was presented with either an underdog brand biography incorporating an external disadvantage but high passion and determination or a top-dog brand biography containing an obvious external advantage but relatively lower passion and determination. In the underdog condition, participants were told, “Company A started in a garage with very limited resources and struggled to succeed.” In the top-dog condition, participants were told, “Company A was well-resourced and favored to succeed in the industry without a large amount of effort.”

After reading one of the two narratives, all participants responded to three 7-point attitude scales to provide their perceptions of Company A (1 = very negative, 7 = very positive; 1 = very bad, 7 = very good; 1 = unfavorable, 7 = favorable) (Batra and Ahtola, 1991; Folkes and Kamins, 1999). Scores on these three items were averaged to form a composite attitude score at time 1 (i.e., ATT_*t*__1_; α = 0.94). Then, both narratives were tested for their level of passion and determination and external disadvantage to check the manipulation of the brand biography (Paharia et al., 2011): “Company A has passion and determination” and “Company A has restrictions from external disadvantage” (1 = strongly disagree, 7 = strongly agree).

Next, all of the respondents were presented with one of two transgression scenarios (Supplementary Appendix A) in which an ethical or functional transgression occurred. Both narratives used a fictitious brand name, “Company A,” but the transgressions were based on actual corporate transgressions committed by real companies in the marketplace. When the company was described as violating ethical norms (adapted from Grappi et al., 2013), participants were told, “Company A didn’t guarantee freedom or minimum rights for their workers.” In the functional transgression condition (adapted from Aaker et al., 2004), participants were told, “Company A’s product malfunction damaged the photos of its consumers.” After reading the narratives, participants responded to the same three 7-point attitude scales. Scores on these three items were averaged to form a composite attitude score at time 2 (i.e., ATT_*t*__2_; α = 0.93). Finally, the manipulation check for transgression type was done: “This transgression is related to individual freedoms or rights,” and “This transgression is related to the company’s technological capacity.” Both statements were assessed on a 7-point scale (1 = not at all, 7 = very much).

### Results

#### Manipulation Checks

A 2 × 2 ANOVA on the manipulation check for brand biography showed that participants who read the underdog scenario marked higher scores on both passion and determination [*M*_*underdog*_ = 6.21 vs. *M*_*top–dog*_ = 2.70; *F*(1, 98) = 471.38, *p* < 0.001] and external disadvantage [*M*_*underdog*_ = 5.42 vs. *M*_*top–dog*_ = 3.46; *F*(1, 98) = 47.25, *p* < 0.001]. The same ANOVA for transgression type also confirmed that participants perceived the transgression scenarios as intended. The ethical transgression was perceived as more related to individual freedoms or rights than the functional one [*M*_*ethical*_ = 5.56 vs. *M*_*functional*_ = 3.06; *F*(1, 98) = 72.77, *p* < 0.001]. The functional transgression scenario was perceived as more related to technological capacity than the ethical one [*M*_*functional*_ = 5.02 vs. *M*_*ethical*_ = 2.69; *F*(1, 98) = 63.20, *p* < 0.001].

#### Attitude Change

**FIGURE 2:**
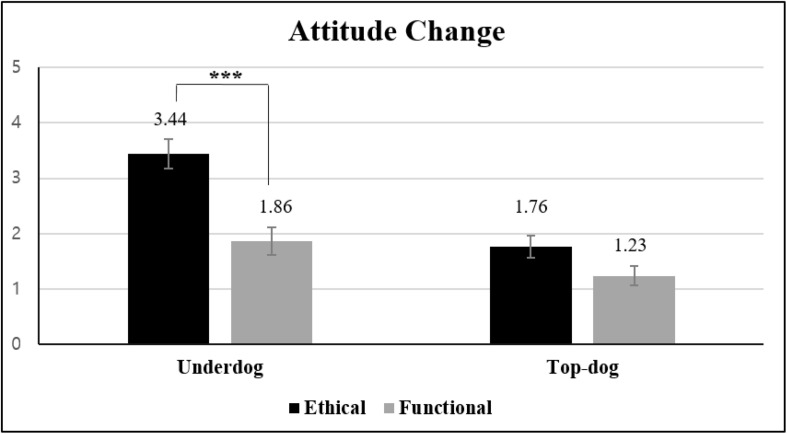
Effects of transgression type and brand biography on attitude change (Study 1). Error bars show the 95% confidence intervals around the means. ***p < 0.001.

In Study 1, we found that the baseline brand attitude for the highly identified underdog was significantly greater than for the top dog [*M*_*underdog*_ = 5.91 vs. *M*_*top–dog*_ = 3.60; *F*(1, 58) = 67.68, *p* < 0.001]. Thus, measuring not only attitude after the transgression but also the baseline brand attitude should provide a more accurate representation of the effect of the transgressions on the brands. Thus, brand attitude was measured twice – both before and after the transgression information was given to the participants. The difference in the two scores on the attitude scale, or ATT_*t*__1_ – ATT_*t*__2_, was used as a dependent measure for analysis. We interpreted the negative change in brand attitude as follows: the higher the score, the severer the impact of the transgressions. A 2 × 2 ANOVA on attitude change indicated that the main effects of transgression type [*M*_*ethical*_ = 2.60 vs. *M*_*functional*_ = 1.56; *F*(1, 98) = 21.04, *p* < 0.001, η*_*p*_*^2^ = 0.177] and brand biography were both significant [*M*_*underdog*_ = 2.59 vs. *M*_*top–dog*_ = 1.49; *F*(1, 98) = 24.90, *p* < 0.001, η*_*p*_*^2^ = 0.203]. More importantly, the two-way interaction was significant [*F*(1, 98) = 5.20, *p* < 0.05, η*_*p*_*^2^ = 0.05]. Planned contrast indicated that attitude changed significantly only for the underdog brand as a function of transgression type [*M*_*ethical*_ = 3.44 vs. *M*_*functional*_ = 1.86; *t*(98) = 4.27, *p* < 0.001]. For the top-dog brand [*M*_*ethical*_ = 1.76 vs. *M*_*functional*_ = 1.23; *t*(98) = 1.62, *p* > 0.109], however, attitude did not vary depending on transgression type (Figure 2).

### Discussion

As predicted, we found that compared with the top-dog brand, participants evaluated the underdog brand more harshly when it was engaged in ethical (vs. functional) transgressions. That is, people had a much stricter standard for the underdog than for the top-dog brand when it came to ethical transgressions. Study 2 was designed to find the underlying mechanism of the ethical underdog trap effect observed in Study 1. We predicted that this effect would be mediated by perceived betrayal (H2).

## Study 2: The Mediating Role of Perceived Betrayal

### Materials and Methods

#### Participants, Design, and Procedure

One hundred eleven undergraduates from a major university in South Korea (*M*_*age*_ = 24.47, *SD* = 3.51; 71 females) completed an online survey through the Qualtrics interface. Participants were randomly assigned to one of four conditions in a 2 (transgression type: ethical vs. functional) × 2 (brand biography: underdog vs. top dog) between-participants design. All procedures in this experiment were the same as in Study 1, except that we measured participants’ level of perceived betrayal. After reading the transgression scenarios, participants provided their ratings of perceived betrayal on four 7-point scale items. Participants assessed the extent to which they felt “loss of confidence,” “betrayal,” “disappointment,” and “loss of trust” (1 = strongly disagree, 7 = strongly agree) (adapted from Grégoire and Fisher, 2008).

### Results

#### Manipulation Checks

A 2 × 2 ANOVA on the manipulation check for brand biography indicated that participants who read the underdog scenario gave higher scores for both passion and determination [*M*_*underdog*_ = 6.11 vs. *M*_*top–dog*_ = 2.70; *F*(1, 107) = 318.26, *p* < 0.001] and external disadvantage [*M*_*underdog*_ = 5.35 vs. *M*_*top–dog*_ = 3.52; *F*(1, 107) = 43.78, *p* < 0.001]. A parallel ANOVA confirmed that the manipulation of transgression type was successful as intended. Each of the transgression scenarios received a higher score on its corresponding manipulation checks [*M*_*ethical*_ = 5.82 vs. *M*_*functional*_ = 3.16; *F*(1, 107) = 114.48, *p* < 0.001 for ethical transgression check; *M*_*functional*_ = 5.71 vs. *M*_*ethical*_ = 2.75; *F*(1, 107) = 124.24, *p* < 0.001 for functional transgression check].

#### Attitude Change

**FIGURE 3:**
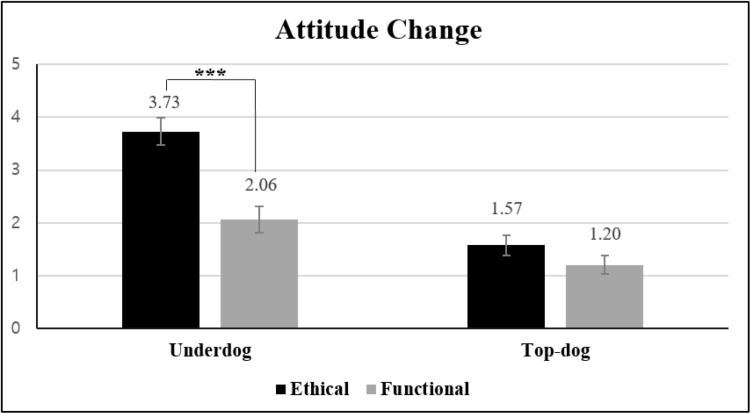
Effects of transgression type and brand biography on attitude change (Study 2). Error bars show the 95% confidence intervals around the means. ***p < 0.001.

A 2 × 2 ANOVA on attitude change [ATT_*t*__1_ – ATT_*t*__2_; α(ATT_*t*__1_) = 0.89, α(ATT_*t*__2_) = 0.93] revealed that the main effects of transgression type [*M*_*ethical*_ = 2.67 vs. *M*_*functional*_ = 1.64; *F*(1, 107) = 27.58, *p* < 0.001, η*_*p*_*^2^ = 0.205] and brand biography were both significant [*M*_*underdog*_ = 2.88 vs. *M*_*top–dog*_ = 1.38; *F*(1, 107) = 60.42, *p* < 0.001, η*_*p*_*^2^ = 0.361]. More critically, the two-way interaction was significant [*F*(1, 107) = 11.18, *p* = 0.001, η*_*p*_*^2^ = 0.095]. Consistent with the results from Study 1, a more negative attitude change was observed only for the underdog brand involving the ethical (vs. functional) transgression [*M*_*ethical*_ = 3.73 vs. *M*_*functional*_ = 2.06; *t*(107) = 6.16 *p* < 0.001]. For the top-dog brand [*M*_*ethical*_ = 1.57 vs. *M*_*functional*_ = 1.20; *t*(107) = 1.33, *p* > 0.186], there was no significant difference in attitude change between the two transgression types (Figure 3).

#### Mediation Analysis

To test whether perceived betrayal (Grégoire and Fisher, 2008) mediated the moderating effect of brand biography on negative attitude change, we employed a bootstrapping analysis using the PROCESS 3.0 macro (model 8) with 5,000 resamples (Hayes, 2017). The model uses transgression type as the independent variable (1 = ethical, 0 = functional), brand biography as the moderator (1 = underdog, 0 = top dog), perceived betrayal (α = 0.81) as the proposed mediator, and attitude change as the dependent variable. The overall mediation effect of perceived betrayal was found to be significant [95% CI = (0.09, 0.72)]. In addition, the conditional indirect effect of transgression type on attitude change was only significant for the underdog condition [95% CI = (0.13, 0.68)], but not for the top-dog condition [95% CI = (-0.15, 0.22)] as reported in Table 2.

**TABLE 2:** Mediation analysis results for Study 2.

	Dependent variables					
	Regression 1 Perceived betrayal			Regression 2 Attitude change		
	*B*	*t*	*p*	*B*	*t*	*p*
Intercept	5.0093	28.0023	<0.0001	-0.2381	-0.4324	0.6664
Transgression type [X]	0.0926	0.3660	0.7151	0.3438	1.2732	0.2057
Brand biography [W]	-0.2075	-0.8349	0.4057	0.9194	3.4558	0.0008
X × W	1.2396	3.5110	0.0007	0.9431	2.3711	0.0195
Perceived betrayal [M]				0.2866	2.7790	0.0065
Regression model	*F*(3, 107) = 12.33, *p* = 0.0007 *R*^2^ = 0.2441			*F*(4, 106) = 28.23, *p* < 0.0001 *R*^2^ = 0.7182		

**Transgression type (1 = ethical vs. 0 = functional); Brand biography (1 = underdog brand vs. 0 = top-dog brand).**

### Discussion

The results of Study 1 were replicated in Study 2. Consistent with H1, we confirmed that participants evaluated the underdog brand more harshly for ethical (vs. functional) transgressions. Furthermore, supporting H2, we corroborated that perceived betrayal mediated the moderating effect of brand biography on the asymmetrical pattern in attitude change between the two transgression types (H1).

## Study 3: A Boundary Condition of the Ethical Underdog Trap

Study 3 was designed to mainly focus on breaking down ethical transgressions into the subtypes of autonomy- and community-related transgressions. We argued that the underdog positioning would not have disadvantages in all of the ethical domains. As conceptualized, underdogs are less likely to be responsible for community-related transgressions involving social ethics (Grappi et al., 2013).

### Materials and Methods

#### Participants, Design, and Procedure

One hundred one undergraduates (*M*_*age*_ = 24.05, *SD* = 3.80; 68 females) recruited through a platform of a major research company in South Korea completed an online survey. Participants were randomly assigned to one of four conditions in a 2 (ethical transgression type: autonomy vs. community) × 2 (brand biography: underdog vs. top dog) between-participants design. This study followed the same procedure as those of the previous studies. For the autonomy-related ethical transgression scenario, Company A was described as violating its workers’ freedoms and human dignity. In the community-related ethical transgression, Company A was portrayed as a greedy company that entered a community and intimidated the livelihood of local shopkeepers (Supplementary Appendix A).

### Results

#### Manipulation Checks

A 2 × 2 ANOVA on the manipulation check for brand biography revealed that both passion and determination [*M*_*underdog*_ = 6.05 vs. *M*_*top–dog*_ = 2.51; *F*(1, 97) = 287.58, *p* < 0.001] and external disadvantage [*M*_*underdog*_ = 5.45 vs. *M*_*top–dog*_ = 3.39; *F*(1, 97) = 35.23, *p* < 0.001] were higher for the participants who read the underdog scenario. A parallel ANOVA on the manipulation check for the types of ethical transgressions demonstrated that a difference existed in the violation of human dignity and freedom [*M*_*autonomy*_ = 5.46 vs. *M*_*community*_ = 4.24; *F*(1, 97) = 13.39, *p* < 0.001]. As we predicted, there was no difference in whether the scenario was related to company ability or product function [*M*_*autonomy*_ = 2.88 vs. *M*_*community*_ = 2.53; *F*(1, 97) = 1.92, *p* = 0.169].

#### Attitude Change

**FIGURE 4:**
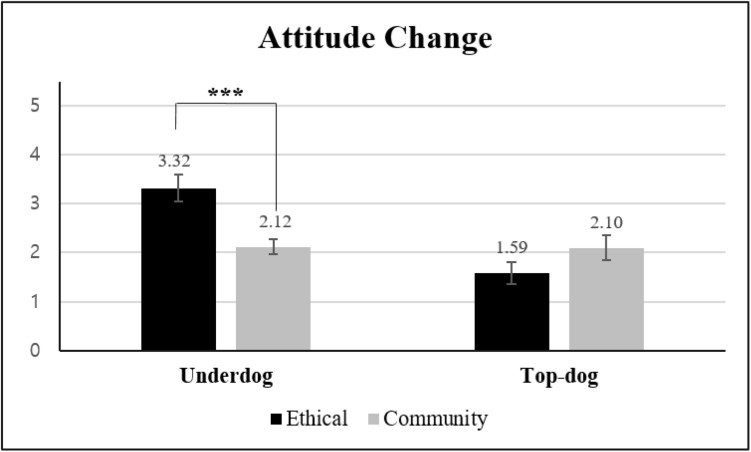
Effects of transgression type and brand biography on attitude change (Study 3). Error bars show the 95% confidence intervals around the means. ***p < 0.001.

A 2 × 2 ANOVA on attitude change [α(ATT_*t*__1_) = 0.89, α(ATT_*t*__2_) = 0.95] indicated that the main effect was only significant for brand biography [*M*_*underdog*_ = 2.82 vs. *M*_*top–dog*_ = 1.84; *F*(1, 97) = 12.25, *p* < 0.001, η*_*p*_*^2^ = 0.112]. More importantly and more relevant to our hypothesis, the two-way interaction was found to be significant [*F*(1, 97) = 11.70, *p* = 0.001, η*_*p*_*^2^ = 0.108]. Planned contrast indicated that attitude changed more negatively for the underdog brand in terms of autonomy-related (vs. community-related) transgressions [*M*_*autonomy*_ = 3.32 vs. *M*_*community*_ = 2.12; *t*(97) = 3.74, *p* < *0.001*]. For the top-dog brand, the subtypes of ethical transgressions [*M*_*autonomy*_ = 1.59 vs. *M*_*community*_ = 2.10; *t*(97) = 1.33, *p* = 0.186] did not significantly influence attitude change (Figure 4).

### Discussion

Building on the first two studies’ findings, Study 3 explored the role of the two subtypes of ethical transgressions. As we predicted, a stricter moral standard for the underdog was only applied for the autonomy-related transgressions. Only the underdog brand was evaluated harshly for autonomy-related (vs. community-related) transgressions, consistent with H3.

## Study 4: The Ethical Underdog Trap in an Integrated Design

This study was designed to integrate the three transgression types (one functional and two ethical) covered in the three previous studies. In this study, we tested all three hypotheses in a single design integrating the three transgression types and the proposed mediator of perceived betrayal.

### Materials and Methods

#### Manipulation Checks

For a more rigorous manipulation of the transgressions, we recruited manipulation check group to determine whether the scenarios were manipulated as we intended. We recruited 30 U.S. residents (*M*_*age*_ = 33.97, *SD* = 14.16; 12 females) from Prolific Academic^1^ and randomly assigned them to one of three conditions in the autonomy, community, and functional scenarios. The manipulation check items included the following: “This transgression is related to individual freedom or rights,” “This transgression is related to community norms,” and “This transgression is related to technological capacity” (1 = not at all and 7 = very much). A one-way ANOVA on the first manipulation check item indicated that there was significant difference among the three transgression types [*M*_*autonomy*_ = 4.73 vs. *M*_*community*_ = 2.73 vs. *M*_*functional*_ = 3.50; *F*(2, 27) = 3.842, *p* = 0.034]. Planned contrast [(1, -1/2, -1/2)] indicated that the autonomy scenario had a significantly higher score compared to the other two scenarios [*t*(27) = 2.14, *p* < *0.05*]. A one-way ANOVA on the second manipulation check verified that there was a significant difference among the three transgression types [*M*_*autonomy*_ = 4.64 vs. *M*_*community*_ = 5.36 vs. *M*_*functional*_ = 3.25; *F*(2, 27) = 4.11, *p* < 0.05]. Planned contrast showed that the community scenario received a significantly higher score compared to the other two [*t*(27) = 2.14, *p* < *0.05*]. For the last check, an ANOVA on the functional transgression indicated that there was significant difference among the three transgression types [*M*_*autonomy*_ = 1.73 vs. *M*_*community*_ = 3.64 vs. *M*_*functional*_ = 6.63; *F*(2, 27) = 56.46, *p* < 0.0001]. Planned contrast [(1, -1/2, -1/2)] showed that the functional scenario had a significantly higher score than the other two scenarios [*t*(27) = 9.62, *p* < *0.0001*].

#### Participant, Design, and Procedure

One hundred twenty U.S. participants (*M*_*age*_ = 35.23, *SD* = 12.19; 58 females)^2^ were recruited *via* Prolific Academic (see text footnote 1). Participants were randomly assigned to one of six conditions in a 3 (transgression type: autonomy vs. community vs. functional) × 2 (brand biography: underdog vs. top dog) between-participants design. All procedures of this study followed those of the previous studies.

### Results

#### Manipulation Checks

A 3 × 2 ANOVA on the manipulation check for brand biography revealed that both passion and determination [*M*_*underdog*_ = 6.42 vs. *M*_*top–dog*_ = 2.40; *F*(2, 114) = 297.28, *p* < 0.001] and external disadvantage [*M*_*underdog*_ = 4.74 vs. *M*_*top–dog*_ = 2.25; *F*(2, 114) = 93.33, *p* < 0.001] were higher for the participants who read the underdog (vs. top-dog) scenario.

#### Attitude Change

**FIGURE 5:**
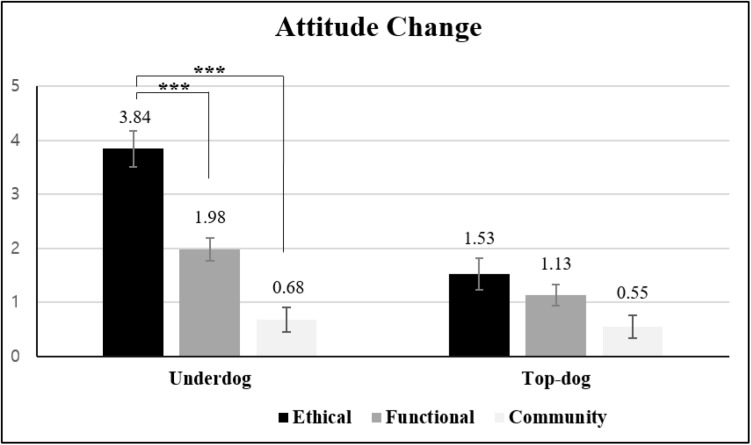
Effects of transgression type and brand biography on attitude change (Study 4). Error bars show the 95% confidence intervals around the means. ***p < 0.001.

A 3 × 2 ANOVA on attitude change [α(ATTt_1_) = 0.87, α(ATT_*t*__2_) = 0.98] indicated that both the main effects of transgression type [*M*_*autonomy*_ = 2.72 vs. *M*_*community*_ = 0.62 vs. *M*_*functional*_ = 1.49; *F*(2, 114) = 24.32, *p* < 0.0001, η*_*p*_*^2^ = 0.299] and brand biography [*M*_*underdog*_ = 2.23 vs. *M*_*top–dog*_ = 1.07; *F*(2, 114) = 20.26, *p* < 0.0001, η*_*p*_*^2^ = 0.151] were significant. More importantly and more relevant to our hypothesis, the two-way interaction was significant [*F*(2, 114) = 6.97, *p* = 0.001, η*_*p*_*^2^ = 0.109]. The planned contrast indicated that for the underdog brand, attitude changed more negatively in response to the autonomy-related transgression than to the functional transgression and the community-related transgression [*M*_*autonomy*_ = 3.84 vs. *M*_*communiy*_ = 0.68 vs. *M*_*functional*_ = 1.98; *t*(114) = 6.87, *p* < *0.001* for the contrast (1, -1/2, -1/2)]. For the top-dog brand, negative attitude change for the autonomy-related transgression was not significantly higher than for the other transgression types [*M*_*autonomy*_ = 1.53 vs. *M*_*communiy*_ = 0.55 vs. *M*_*function*_ = 1.13; *t*(114) = 1.92, *p* > *0.057* for the contrast (1, -1/2, -1/2)] as reported in Figure 5.

#### Mediation Analysis

To explain the process of the observed results, we used the bootstrapping PROCESS 3.0 macro (model 8) with 5,000 resamples (Hayes, 2017) to test for moderated mediation. The three transgression types of the multi-categorical independent variable were coded with an indicator coding system [transgression type as the independent variable (X_1_, X_2_): autonomy (0, 0), community X_1_ = (1, 0), X_2_ = functional (0, 1); brand biography as the moderator: 1 = underdog, 0 = top dog]. The overall moderated mediation effect of perceived betrayal was found to be significant [X_1_: 95% CI = (0.22, 1.38); X_2_: 95% CI = (0.00, 1.07)]. Moreover, both the conditional indirect effects of transgression type on attitude change were only significant for the underdog condition [X_1_: 95% CI = (0.42, 1.63); X_2_: 95% CI = (0.16, 1.11)] but not for the top-dog condition [X_1_: 95% CI = (-0.07, 0.62); X_2__:_ 95% CI = (-0.18, 0.42)] as reported in Table 3.

**TABLE 3:** Mediation analysis results for Study 4.

	Dependent variables					
	Regression 1 Perceived betrayal			Regression 2 Attitude change		
	*B*	*t*	*p*	*B*	*t*	*p*
Intercept	5.5250	18.0080	<0.0001	−0.3421	−0.6237	0.5341
Transgression type [X_1_]	−0.6750	−1.5557	0.1226	−0.7542	−1.8867	0.0618
Transgression type [X_2_]	−0.3837	−0.9146	0.3623	−0.2727	−0.7103	0.4790
Brand biography [W]	0.5821	1.3579	0.1772	2.1103	5.3564	0.0000
X_1_ × W	−2.0637	−3.3611	0.0011	−1.4732	−2.5103	0.0135
X_2_ × W	−0.7529	−1.2272	0.2223	−1.2024	−2.1358	0.0349
Perceived betrayal [M]				0.3394	3.9753	0.0001
Regression model	*F*(5, 114) = 8.79, *p* < 0.0001 *R*^2^ = 0.5274			*F*(6, 113) = 18.72, *p* < 0.0001 *R*^2^ = 0.7060		

**Transgression type (X_1_, X_2_) = [autonomy {0, 0}, community {1, 0}, functional {0, 1}]; Brand biography (1 = underdog brand vs. 0 = top-dog brand).**

### Discussion

Utilizing a single research design, we replicated the findings of the previous studies by exploring the whole picture. As was observed before, transgression type was only meaningful for the underdog brand positioning. The autonomy-related transgression damaged the underdog brand more seriously than both the community-related (H3) and functional (H1) transgressions. In particular, the moderated mediation effect of perceived betrayal emerged as the same underlying mechanism that produced the significant differences among the three transgression conditions (H2).

## General Discussion

The underdog brand appeal relates more to value than utilitarian benefits. The underdog brand that is short on resources but highly identified by most consumers and has a passionate heart is considered an attractive branding position that captures public favor. People may root for the underdog brand and get vicarious satisfaction when it succeeds. Thus, the underdog positioning is powerful enough to create a positive attitude toward the brand (McGinnis and Gentry, 2009; Paharia et al., 2011; Kao, 2015; McGinnis et al., 2017). However, in this research, we warn that the underdog positioning can be a piece of cheese in the trap when ethical transgressions occur.

To demonstrate the ethical underdog trap effect, we examine the situation in which the underdog brand positioning backfires. By applying the information matching-and-mismatching principle (Anderson, 1981; Edwards, 1990; Petty and Wegener, 1998; Fabrigar and Petty, 1999), we prove in Study 1 that people become very upset with the underdog brand when the symbolic brand commits value-related ethical transgressions. Thus, the underdog positioning leads to harsher judgment from consumers compared to the top-dog position. In Study 2, the underdog trap effect is explained with perceived betrayal as the underlying mechanism. In Study 3, we suggest a boundary condition for the proposed effect, in which the effect is only validated for autonomy-related ethical transgressions. When community norms are violated, this adverse underdog effect disappears. In Study 4, we replicate the effect of the ethical underdog trap in an integrated design with all three transgression types discussed: two ethical transgressions related to autonomy and community norms and a performance-oriented functional transgression. In this study, we corroborate that the results are consistent with those of Studies 1 through 3. Of particular note, the moderated mediation effect of perceived betrayal is also demonstrated as the underlying mechanism of the underdog trap effect.

### Theoretical Contributions

The findings of this research offer several important contributions to the current understanding of consumer psychology and brand perception after brand crisis. Above all, this is the first study to demonstrate the ethical trap of the underdog brand through bringing to light the dark side of the underdog effect. Since the underdog brand is in an attractive position that connects with consumers’ self-identifying underdog image, the underdog positioning creates a strong empathic appeal in its shared disadvantaged position in society. However, to fully enjoy the advantages of the underdog positioning, a prerequisite condition must be satisfied. Love enjoyed through empathic concern must be accompanied by responsibility. Consumers support underdog brands as extensions of themselves and thus expect them to protect other underdogs’ basic autonomy rights in society. Consumers believe that their support for the underdog brand deserves reciprocation. By introducing the unique prerequisite conditions in the ethical realm, this research makes a meaningful contribution to literature not only concerning the underdog effect, but its dark side as well. Based on the findings, only when ethical responsibilities of the underdog are fulfilled can the underdog positioning achieve favorable attitudes from consumers. When underdog brands are tainted with unethical issues, the consumers’ support may backfire.

Second, the current study classifies not only performance-related transgressions and value-related transgressions (Pullig et al., 2006; Roehm and Brady, 2007; Dutta and Pullig, 2011) but also distinguishes value-related transgressions in detail from autonomy and community-related ethical transgressions (Shweder et al., 1997; Rozin et al., 1999; Grappi et al., 2013). This study gives meaningful theoretical implications to both marketing and psychology literature by proposing the interaction effects of different categories of transgressions and brand positioning. It is worth noting that this is the first attempt covering two sub-ethical transgressions including performance-related transgressions in an integrated model. Hence, current research makes it possible to corroborate separately scattered research results in one joint model. Further, the distinction between the transgression types enriches brand literature, but also human psychology based on consumer perspective.

Moreover, by demonstrating perceived betrayal as a psychological mechanism to understand why the ethical underdog trap effect occurs, we contribute to the literature on customer–brand interactions in the research stream of interpersonal reactivity after brand transgressions (Cleeren et al., 2017). In particular, we extend love-becomes-hate effects (Grégoire and Fisher, 2008) to the underdog positioning brands where customers’ support based on identification with the underdog brands’ symbolism backfired in ethical transgression. In addition, considering the underdog brand in light of symbolic connotation provides opportunity to unfold the search-and-alignment model in the information matching-and-mismatching literature (Petty and Wegener, 1998; Fabrigar and Petty, 1999; Pham and Muthukrishnan, 2002; Pullig et al., 2006). It is presumed that the underdog positioning delivers symbolic benefits rather than practical and immediate advantages (McGinnis and Gentry, 2009; Paharia et al., 2011). However, this is the first empirical study to demonstrate consumers’ perception of underdog brand and ethical expectation. In particular, this study reveals how customers perceive the symbolic aspects of the underdog and further directly demonstrates its effects on the consumers’ moral beliefs toward the underdog accompanying the symbolic brand image.

### Managerial Implications

By thoroughly exploring the ethical underdog trap, this research generates insightful managerial implications. It is worthwhile for marketing managers to understand the nature of the underdog brand positioning, especially in times of brand crisis. Since the underdog brand is symbolic by nature in highlighting the self–brand connection, just providing high-quality products or services is not enough. To satisfy consumers, the brand must have high ethical standards. Thus, when positioning their brands as the underdog, marketers should be cautious not to be involved in moral issues, especially those that threaten laypeople’s basic autonomy rights. Further, this research focused on how consumers could alter their attitudes toward the brands after the transgressions occur. First impressions toward the brand is important, but the brand attitude is rather dynamic. The positive attitudes toward the brands can easily turn negative and vice versa. Based on love-becomes-hate effects, the most loyal customers can be the worst enemies. In other words, because of small or big issues, consumers continually change their attitudes toward the brand and switch to other brands when they lose affection. Although the clear importance lies in identifying the nature of brand attitude changes over time, most of the research in marketing dealing with consumer behavior catches only temporal brand attitudes (Grossman and Till, 1998; He et al., 2016). Based on this research, we learned that the more critical factor is in observing brand attitudes in a continuous timeline. Thus, this research can be a fresh guideline for marketing practitioners in the field. The attitudes toward the brand are dynamic and may even be more vulnerable to the positively perceived brands when consumer expectations are not fulfilled. Moreover, when a highly identified brand with affection ruins the unique expectations of consumers, the affection can backfire (Millar and Millar, 1990).

Second, the current study explored the moderating role of brand biography in the relationship between the brand transgression type and brand attitude change through perceived betrayal. For top-dog brands, the type of transgression involved was not a critical factor in changing a consumer’s attitude toward the brand. However, the participants negatively transformed their attitudes when the underdog brands committed value-related ethical transgressions. Especially when the ethical transgression was autonomy-related, consumers felt highly betrayed and exhibited a negative attitude change. Furthermore, when the ethical transgression was about social responsibility, which is considered a community transgression, it worked as a boundary condition. However, the positive brand attitudes toward the underdog were sustained even after committing a community transgression. The aforementioned moderating role of brand biography and the transgression types with a boundary condition were all replicated in the integrated model of Study 4. Thus, the type of brand transgression in which the underdog is involved may be critical, but how to categorize the transgression and communicate the nature of such transgressions to the consumers may be a more crucial factor in altering consumer attitudes toward the related brands (Ran et al., 2016).

As previously mentioned, when such transgressions occur, the way the brand communicates with its customers is an important factor that can amplify or alleviate the rage of consumers (Kim and Choi, 2016). Based on the current research, perceived betrayal is a key emotion that causes consumers to negatively change their attitudes. As such, marketers should devise ways to mitigate the consumers’ feelings of betrayal. In light of the consumer–brand relationship, simply emphasizing the self–brand connection may backfire. Negative emotions may be amplified because a friend’s betrayal is most bitter of all. Thus, delivering a sincere, personal message may be the best way (Bachman and Guerrero, 2006; Knight et al., 2015). For example, instead of sending a group e-mail, utilizing direct and personal apologies to consumers may relieve negative consequences of autonomy-related ethical transgressions. In addition, in the real marketing field, erasing the brand transgression in the consumers’ mind might be impossible. However, for marketing managers, categorizing brand transgression can be possible. Thus, the current study suggests that rather than trying to suppress the degree of brand transgression issues, attempting to categorize the transgression type could alleviate consumers’ betrayal. As for the underdog brand, not all ethical transgressions increased consumer betrayal. Only specific transgressions categorized as autonomy-related critically altered brand attitudes in a negative direction. However, since customers have a relatively high tolerance concerning community-related ethical and functional transgressions, underdog brands can benefit from emphasizing the self–brand connection in communications if these two types of transgressions occur. This benefit is clearly a positive buffering effect of the underdog position in contrast to the ethical underdog trap effect.

### Limitation and Further Research

First, consumer identification with underdog brands is likely limited to certain segments of consumers. For instance, numerous cultural and individual differences may suggest that certain consumers – for example, those who are high in power distance belief or in social dominance orientation – may not identify with the underdog. However, the underdog effects based on highlighting psychological mechanisms with identification have been demonstrated in various studies; thus, the underdog effect is quite robustly acceptable (McGinnis and Gentry, 2009; Paharia et al., 2011; Kao, 2015; Kirmani et al., 2017; McGinnis et al., 2017; Kim et al., 2019). Also, it is important to distinguish the desire to be a top dog from top-dog identification. Some people might want to be a top dog, but this desire is different from identifying with a top dog. Based on previous underdog literature, people normally perceive themselves as the underdog (McGinnis and Gentry, 2009; Paharia et al., 2011; Kao, 2015). However, there might be some outliers who identify with the top dog. Although, even including these outliers, we found statistically significant results to support our hypotheses. Therefore, this limitation may not be a critical factor affecting this study. Further, while some people may not currently identify with the underdog, the underdog brand can be a reminder of the consumer’s past underdog story. In a future study, a more specific path for people’s identification with the underdog could be explored.

The main dependent variable for the current study, attitude change to brands, leaves room for further research. Among the four studies, three studies indicate that significantly higher attitude toward the underdog brands compared to top dog decreased as insignificant after ethical transgression (see Supplementary Appendix B). However, ratings for underdog brands were still higher than top-dog brands. Thus, further research can explore stronger effects to observe reversed underdog effect by considering different dependent variables such as purchase intention. In addition, to provide more realistic implications, the authors will design field studies by applying real brands and situation.

Although we suggested and demonstrated perceived betrayal as the psychological mechanism in the current study, an alternative explanation could instead be proposed, such as other negative emotions. However, for the current study, we believe that perceived betrayal truly explains the underlying process of the ethical underdog trap. Compared to the most possible alternative, such as anger, perceived betrayal is unique in that it is experienced within the context of a relationship. Betrayal, which is based on human relationships, is distinguished from emotions like dissatisfaction and anger (Smith et al., 1999; Bougie et al., 2003); unlike these other emotions that can exist outside of the relationship context, betrayal is associated with the standards that govern relationships. Since underdog effects are rooted in the relationship between the brand and the consumer who identifies with the brand, theoretically, perceived betrayal is appropriate to explain this phenomenon and the empirical studies we conducted also demonstrated this point.

Further, we admit the small sample size as a limitation of the current study. Given the limited access to the participant pool from Studies 1–3, we collected responses from all the students who signed up to complete the online experiment, rather than determining an *a priori* sample size. In Study 4, we consider consistency as a priority when collecting the participants to encapsulate all of the four studies in an integrated research model. Further, to supplement this limitation, we additionally conducted a single-paper meta-analysis (McShane and Böckenholt, 2017), which revealed that an single-paper meta-analysis (SPM) of our studies estimates the first effect at 1.56 (95% CI: 0.95, 2.17). The confidence interval did not contain zero, thus demonstrating the robustness of our findings and indicating that participants changed their attitudes more negatively when underdog brands committed ethical vs. functional transgression. However, the second effect was estimated at 0.39 (95% CI: -0.17, 0.95). The confidence interval did include zero, thus indicating that the transgression type did not affect attitude change toward top-dog brands (see Supplementary Appendix C). Finally, to make up for this small sample limitation, the authors will replicate the current study with broader and international samples in further research.

## Conclusion

Intentional or not, brands commit transgressions that throw them into crisis. Some transgressions are considered trivial, but others may lead to fatal consequences for the brand. For the symbolic underdog brand, ethical transgressions prove to be deadly serious because consumers’ moral standard for the underdog brand is particularly high. Thus, when underdog brands are tainted with ethical issues, consumers feel betrayed and negatively change their attitudes. Social support demands responsibility. Underdog brands should recognize that consumer support may become a trap if ethical responsibility is not fulfilled.

## Data Availability Statement

The datasets generated for this study are available on request to the corresponding author.

## Ethics Statement

The studies involving human participants were reviewed and approved by the Seoul National University. The patients/participants provided their written informed consent to participate in this study.

## Author Contributions

YK and KP jointly designed, analyzed, and wrote this manuscript, and undertook all the testing and data collection. All authors contributed to the article and approved the submitted version.

## Conflict of Interest

The authors declare that the research was conducted in the absence of any commercial or financial relationships that could be construed as a potential conflict of interest.

## Footnotes

1 www.prolific.ac

2 Given the limited access to the participant pool from the Study 1–3, we collected responses from all the students who signed up to complete the online experiment, rather than determining an *a priori* sample size. A *post-hoc* power analysis in G^∗^Power indicated that the sample size provides Study1 = 71%, Study 2 = 74%, and Study 3 = 70% power to detect effects (with a *F*-test, ANOVA: main effects and interactions; effect size *F* = 0.025, α err prob = 0.05). Thus, in Study 4 we consider consistency as a priority when collecting the participants to encapsulate all of the four studies in an integrated research model. Thus, we collected participants for Study 4 which can provide similar power with previous studies; the *post-hoc* power analysis of Study 4 sample size provides 68% detect effects.

## References

[B1] Aaker J. Fournier S. Brasel S. A. (2004). When good brands do bad. *J. Consumer Res.* 31 1–16. 10.1086/383419

[B2] Anderson N. H. (1981). *Foundations of Information Integration Theory.* New York, NY: Academic Press.

[B3] Bachman G. F. Guerrero L. K. (2006). Forgiveness, apology, and communicative responses to hurtful events. *Commun. Rep.* 19 45–56. 10.1080/08934210600586357

[B4] Batra R. Ahtola O. T. (1991). Measuring the hedonic and utilitarian sources of consumer attitudes. *Mark. Lett.* 2 159–170. 10.1007/BF00436035

[B5] Berens G. van Riel C. B. M. van Bruggen G. H. (2005). Corporate associations and consumer product responses: the moderating role of corporate brand dominance. *J. Mark.* 69 35–48. 10.1509/jmkg.69.3.35.66357 11670861

[B6] Bernstein W. M. Davis M. H. (1982). Perspective-taking, self-consciousness, and accuracy in person perception. *Basic. Appl. Soc. Psychol.* 3 1–19. 10.1207/s15324834basp0301_1

[B7] Bhat S. Reddy S. K. (1998). Symbolic and functional positioning of brands. *J. Consum. Mark.* 15 32–43. 10.1108/07363769810202664

[B8] Bhattacharya C. B. Sen S. (2003). Consumer-company identification: a framework for understanding consumers’ relationships with companies. *J. Mark.* 67 76–88. 10.1509/jmkg.67.2.76.18609 11670861

[B9] Blau P. (1962). *Exchange and Power in Social Life.* New York, NY: Wiley.

[B10] Boehm C. Boehm C. (2009). *Hierarchy in the Forest: The Evolution of Egalitarian Behavior.* Cambridge, MA: Harvard University Press.

[B11] Bougie R. Pieters R. Zeelenberg M. (2003). Angry customers don’t come back, they get back: the experience and behavioral implications of anger and dissatisfaction in services. *J. Acad. Mark. Sci.* 31 377–393. 10.1177/0092070303254412

[B12] Brockner J. Tyler T. R. Cooper-Schneider R. (1992). The influence of prior commitment to an institution on reactions to perceived unfairness: the higher they are, the harder they fall. *Adm. Sci.* 37 241–261. 10.2307/2393223

[B13] Brunk K. H. de Boer C. (2018). How do consumers reconcile positive and negative CSR-related information to form an ethical brand perception? A mixed method inquiry. *J. Bus. Ethics* 161 443–458. 10.1007/s10551-018-3973-3974

[B14] Cialdini R. B. Borden R. J. Thorne A. Walker M. R. Freeman S. Sloan L. (1976). Basking in reflected glory: three (football) field studies. *J. Pers. Soc. Psychol.* 34 366–375. 10.1037/0022-3514.34.3.366

[B15] Cialdini R. B. de Nicholas M. E. (1989). Self-presentation by association. *J. Pers. Soc. Psychol.* 57 626–631. 10.1037/0022-3514.57.4.626

[B16] Cialdini R. B. Richardson K. D. (1980). Two direct tactics of impression management: basking and blasting. *J. Pers. Soc. Psychol.* 39 406–415. 10.1037//0022-3514.39.3.406

[B17] Cleeren K. Dekimpe M. G. van Heerde H. J. (2017). Marketing research on product-harm crises: a review, managerial implications, and an agenda for future research. *J. Acad. Mark. Sci.* 45 593–615. 10.1007/s11747-017-0558-551

[B18] Davis M. H. (1983). Measuring individual differences in empathy: evidence for a multidimensional approach. *J. Pers. Soc. Psychol.* 44 113–126. 10.1037/0022-3514.44.1.113

[B19] Dawar N. Pillutla M. M. (2000). Impact of product-harm crises on brand equity: the moderating role of consumer expectations. *J. Mark. Res.* 37 215–226. 10.1509/jmkr.37.2.215.18729 11670861

[B20] Dutta S. Pullig C. (2011). Effectiveness of corporate responses to brand crises: the role of crisis type and response strategies. *J. Bus. Res.* 64 1281–1287. 10.1016/j.jbusres.2011.01.013

[B21] Edwards K. (1990). The interplay of affect and cognition in attitude formation and change. *J. Pers. Soc. Psychol.* 59 202–216. 10.1037/0022-3514.59.2.202

[B22] Elangovan A. R. Shapiro D. L. (1998). Betrayal of trust in organizations. *Acad. Manag. Rev.* 23 547–566. 10.2307/259294

[B23] Fabrigar L. R. Petty R. E. (1999). The role of the affective and cognitive bases of attitudes in susceptibility to affectively and cognitively based persuasion. *Pers. Soc. Psychol. Bull.* 25 363–381. 10.1177/0146167299025003008

[B24] Finkel E. J. Rusbult C. E. Kumashiro M. Hannon P. A. (2002). Dealing with betrayal in close relationships: does commitment promote forgiveness? *J. Pers. Soc. Psychol.* 82 956–974. 10.1037/0022-3514.82.6.956 12051583

[B25] Folkes V. Kamins M. (1999). Effects of information about firms’ ethical and unethical actions on consumers’ attitudes. *J. Cons. Psychol.* 8 243–259. 10.1207/s15327663jcp0803_03 26627889

[B26] Fournier S. (1998). Consumers and their brands: developing relationship theory in consumer research. *J. Cons. Res.* 24 343–353. 10.1086/209515

[B27] Gaustad T. Samuelsen B. M. Warlop L. Fitzsimons G. J. (2019). Too much of a good thing? consumer response to strategic changes in brand image. *Int. J. Res. Mark.* 36 264–280. 10.1016/j.ijresmar.2019.01.001

[B28] Goldschmied N. Galily Y. Keith K. (2018). Evidence for cross-cultural support for the underdog: is the affiliation driven by fairness and competence assessments? *Front. Psychol.* 9:2246. 10.3389/fpsyg.2018.02246 30510532 PMC6254058

[B29] Grappi S. Romani S. Bagozzi R. P. (2013). Consumer response to corporate irresponsible behavior: moral emotions and virtues. *J. Bus. Res.* 66 1814–1821. 10.1016/j.jbusres.2013.02.002

[B30] Grégoire Y. Fisher R. J. (2008). Customer betrayal and retaliation: when your best customers become your worst enemies. *J. Acad. Mark. Sci.* 36 247–261. 10.1007/s11747-007-0054-50

[B31] Grossman R. P. Till B. D. (1998). The persistence of classically conditioned brand attitudes. *J. Advert.* 27 23–31. 10.1080/00913367.1998.10673540

[B32] Hayes A. F. (2017). *Introduction to Mediation, Moderation, and Conditional Process Analysis: A Regression-Based Approach.* New York, NY: Guilford Publications.

[B33] He Y. Chen Q. Alden D. L. (2016). Time will tell: managing post-purchase changes in brand attitude. *J. Acad. Mark. Sci.* 44 791–805. 10.1007/s11747-015-0444-447

[B34] Hedrick N. Beverland M. Minahan S. (2007). An exploration of relational customers’ response to service failure. *J. Serv. Mark.* 21 64–72. 10.1108/08876040710726301

[B35] Kao D. T. (2015). Is Cinderella resurging? The impact of consumers’ underdog disposition on brand preferences: underdog brand biography and brand status as moderators. *J. Consum. Behav.* 14 307–316. 10.1002/cb.1521

[B36] Kim S. Choi S. M. (2016). Congruence effects in post-crisis CSR communication: the mediating role of attribution of corporate motives. *J. Bus. Ethics* 153 447–463. 10.1007/s10551-016-3425-y

[B37] Kim Y. Park K. Lee S. S. (2019). The underdog trap: the moderating role of transgression type in forgiving underdog brands. *Psychol. Mark.* 36 28–40. 10.1002/mar.21155

[B38] Kirmani A. Hamilton R. W. Thompson D. V. Lantzy S. (2017). Doing well versus doing good: the differential effect of underdog positioning on moral and competent service providers. *J. Mark.* 81 103–117. 10.1509/jm.15.0369 11670861

[B39] Knight J. G. Mather D. Mathieson B. (2015). “The key role of sincerity in restoring trust in a brand with a corporate apology,” in *Marketing Dynamism & Sustainability: Things Change, Things Stay the Same*, ed. Robinson L. Jr. (Cham: Springer), 192–195. 10.1007/978-3-319-10912-1_64

[B40] Koehler J. J. Gershoff A. D. (2003). Betrayal aversion: when agents of protection become agents of harm. *Org. Behav. Hum. Decis. Process.* 90 244–261. 10.1016/S0749-5978(02)00518-516

[B41] Kouchaki M. Dobson K. S. H. Waytz A. Kteily N. S. (2018). The link between self-dehumanization and immoral behavior. *Psychol. Sci.* 29 1234–1246. 10.1177/0956797618760784 29787345

[B42] Kwak H. Puzakova M. Rocereto J. F. (2017). When brand anthropomorphism alters perceptions of justice: the moderating role of self-construal. *Int. J. Res. Mark.* 34 851–871. 10.1016/j.ijresmar.2017.04.002

[B43] Lin C. H. Huang Y. (2018). How self-construals affect responses to anthropomorphic brands, with a focus on the three-factor relationship between the brand, the gift-giver and the recipient. *Front. Psychol.* 9:2070. 10.3389/fpsyg.2018.02070 30455652 PMC6230587

[B44] MacInnis D. J. Folkes V. S. (2017). Humanizing brands: when brands seem to be like me, part of me, and in a relationship with me. *J. Cons. Psychol.* 27 355–374. 10.1016/j.j.2016.12.003

[B45] McCullough M. E. Rachal K. C. Sandage S. J. Worthington E. L. Jr. Brown S. W. Hight T. L. (1998). Interpersonal forgiving in close relationships: II. Theoretical elaboration and measurement. *J. Pers. Soc. Psychol.* 75 1586–1603. 10.3389/fpsyg.2016.01775 9914668

[B46] McGinnis L. P. Gao T. Jun S. Gentry J. W. (2017). Motivational bases for consumers’ underdog affection in commerce. *J. Ser. Manag.* 28 563–592. 10.1108/JOSM-02-2016-2052

[B47] McGinnis L. P. Gentry J. W. (2009). Underdog consumption: an exploration into meanings and motives. *J. Bus. Res.* 62 191–199. 10.1016/j.jbusres.2008.01.026

[B48] McShane B. B. Böckenholt U. (2017). Single-paper meta-analysis: benefits for study summary, theory testing, and replicability. *J. Consum. Res.* 43 1048–1063. 10.1093/jcr/ucw085

[B49] Metts S. (1994). “Relational transgressions,” in *The Dark Side of Interpersonal Communications*, eds Spitzberg B. H. Cupach W. R. (New Jersy: Lawrence Erlbaum), 217–239.

[B50] Millar M. G. Millar K. U. (1990). Attitude change as a function of attitude type and argument type. *J. Pers. Soc. Psychol.* 59 217–228. 10.1037/0022-3514.59.2.217

[B51] Moreland R. L. McMinn J. G. (1999). Gone but not forgotten: loyalty and betrayal among ex-members of small groups. *Pers. Soc. Psychol. Bull.* 25 1476–1486. 10.1177/01461672992510004

[B52] Muthukrishnan A. V. Pham M. T. Mungale A. (1999). Comparison opportunity and judgement revision. *Org. Behav. Hum. Decis. Process.* 80 228–251. 10.1006/obhd.1999.2859 10579964

[B53] Paharia N. Keinan A. Avery J. Schor J. B. (2011). The underdog effect: the marketing of disadvantage and determination through brand biography. *J. Consum. Res.* 37 775–790. 10.1086/656219

[B54] Petty R. E. Wegener D. T. (1998). Matching versus mismatching attitude functions: implications for scrutiny of persuasive messages. *Pers. Soc. Psychol. Bull.* 24 227–240. 10.1177/0146167298243001

[B55] Pham M. T. Muthukrishnan A. V. (2002). Search and alignment in judgment revision: implications for brand positioning. *J. Mark. Res.* 39 18–30. 10.1509/jmkr.39.1.18.18929 11670861

[B56] Piff P. K. Kraus M. W. Côté S. Cheng B. H. Keltner D. (2010). Having less, giving more: the influence of social class on prosocial behavior. *J. Pers. Soc. Psychol.* 99 771–784. 10.1037/a0020092 20649364

[B57] Pullig C. Netemeyer R. G. Biswas A. (2006). Attitude basis, certainty, and challenge alignment: a case of negative brand publicity. *J. Acad. Mark. Sci.* 34 528–542. 10.1177/0092070306287128

[B58] Ran Y. Wei H. Li Q. (2016). Forgiveness from emotion fit: emotional frame, consumer emotion, and feeling-right in consumer decision to forgive. *Front. Psychol.* 7:1775. 10.3389/fpsyg.2016.01775 PMC5109223 27895612

[B59] Roehm M. L. Brady M. K. (2007). Consumer responses to performance failures by high-equity brands. *J. Consum. Res.* 34 537–545. 10.1086/520075

[B60] Roloff M. E. Soule K. P. Carey C. M. (2001). Reasons for remaining in a relationship and responses to relational transgressions. *J. Soc. Pers. Relat.* 18 362–385. 10.1177/0265407501183004

[B61] Rozin P. Lowery L. Imada S. Haidt J. (1999). The CAD triad hypothesis: a mapping between the other-directed moral emotions, disgust, contempt and anger and three universal moral codes. *J. Pers. Soc. Psychol.* 76 574–586. 10.1037/0022-3514.76.4.574 10234846

[B62] Shweder R. A. Much N. C. Mahapatra M. Park L. (1997). “The “big three” of morality (autonomy, community, and divinity) and the “big three” explanations of suffering,” in *Morality and Health*, eds Brandt A. Rozin P. (New York, NY: Routledge), 119–169.

[B63] Sinha J. Lu F. C. (2016). “I” value justice, but “we” value relationships: self-construal effects on post-transgression consumer forgiveness. *J. Consum. Psychol.* 26 265–274. 10.1016/j.jcps.2015.06.002

[B64] Smith A. K. Bolton R. N. Wagner J. (1999). A model of customer satisfaction with service encounters involving failure and recovery. *J. Mark. Res.* 36 356–372. 10.1177/002224379903600305

[B65] Snodgrass S. E. (1985). Women’s intuition: the effect of subordinate role on interpersonal sensitivity. *J. Pers. Soc. Psychol.* 49 146–155. 10.1037/0022-3514.49.1.146

[B66] Teresi M. Pietroni D. D. Barattucci M. Giannella V. A. Pagliaro S. (2019). Ethical Climate (s), Organizational Identification, and Employees’ behavior. *Front. Psychol.* 10:1356. 10.3389/fpsyg.2019.01356 31275196 PMC6593040

[B67] Van den Bos K. Lind E. A. (2002). Uncertainty management by means of fairness judgments. *Adv. Exp. Soc. Psychol.* 34 1–60. 10.1016/S0065-2601(02)80003-X

[B68] Van Prooijen J. W. Lam J. (2007). Retributive justice and social categorizations: the perceived fairness of punishment depends on intergroup status. *Eur. J. Soc. Psychol.* 37 1244–1255. 10.1002/ejsp.421

[B69] Van Vugt M. Hogan R. Kaiser R. B. (2008). Leadership, followership, and evolution: some lessons from the past. *Am. Psychol.* 63 182–196. 10.1037/0003-066X.63.3.182 18377108

[B70] Ward J. C. Ostrom A. L. (2006). Complaining to the masses: the role of protest framing in customer-created complaint web sites. *J. Consum. Res.* 33 220–230. 10.1086/506303

[B71] Waytz A. Cacioppo J. Epley N. (2010). Who sees human? The stability and importance of individual differences in anthropomorphism. *Perspect. Psychol. Sci.* 5 219–232. 10.1177/1745691610369336 24839457 PMC4021380

