# Supplementary material for: When the Underdog Positioning Backfires! The Effects of Ethical Transgressions on Attitudes Toward Underdog Brands
Source: Front Psychol. 2020 Sep 4;11:1988. doi: 10.3389/fpsyg.2020.01988 (PMC7498723; doi:10.3389/fpsyg.2020.01988)

When the Underdog Positioning Backfires! The Effects of Ethical Transgressions on Attitudes Toward Underdog Brand

# Appendix A. Scenario for brand biography and transgression type

| **Brand Biography** | |
| --- | --- |
| Underdog | Top-dog |
| Camera Company A started its business in a garage without an office space. As it was a newly created start-up company, competing with big corporations was extremely challenging. Compared to the competitors, Company A had many disadvantages such as limited resources.  Recently, however, the company invented a new DSLR Camera A12 that outperformed their competitors’ products. This was all possible because of the employees who promoted passion and a challenging spirit in the work place. | Camera Company A created DSLR Camera A12 five years ago and was recognized for its performance and technology throughout the market. Since then, the company has been in the second place in terms of total market share.  However, its employees are now satisfied with the current position in the market. Instead of striving to become number one, the company is willing to remain in its current position and is not developing new products or providing better services to customers. |
| **Brand Transgression** | |
| Ethical Transgression | |
| Autonomy | Community |
| Here is a news summary about Company A:  A whistle-blower has exposed misconduct and illegal activity occurring in Company A. This company has not been protecting the workers’ fundamental rights, especially for temporary, female, and handicapped workers. Moreover, the news media found and reported that the company disabled the labor union. | Here is a news summary about Company A:  Recently, the company acted as a law-abiding investor and acquired all the stores around the factory sites for much lower than the market value. After the acquisition, the company demolished all the local stores and the previous store owners lost everything they had in the end. |
| Functional Transgression | |
| Here is a news summary about Company A:  Recently, the marketing team promoted that the company would restore black-and-white pictures to color pictures, and then would post the photos in the company website’s digital photo album section. This promotion applied to all who had purchased the A12 camera. However, a problem occurred during the restoration process. A malfunction of the alternation machine destroyed all the black-and-white pictures the company received from its customers. | |

# Appendix B. Attitude before and after transgression (Study 1-4)

| Study | Transgression Type | Brand Biography | Before Transgression (ATT_t1_) | *t* | *P* | After Transgression (ATT_t2_) | *t* | *P* |
| --- | --- | --- | --- | --- | --- | --- | --- | --- |
| Study  1 | Ethical | Underdog | 5.94 | 7.710 | .000 | 2.50 | 1.413 | .161 |
|  |  | Top-dog | 3.85 |  |  | 2.08 |  |  |
|  | Functional | Underdog | 5.86 | 7.187 | .000 | 4.00 | 4.378 | .000 |
|  |  | Top-dog | 4.01 |  |  | 2.78 |  |  |
| Study 2 | Ethical | Underdog | 5.98 | 9.71 | .000 | 2.25 | .119 | .906 |
|  |  | Top-dog | 3.79 |  |  | 2.22 |  |  |
|  | Functional | Underdog | 5.72 | 6.62 | .000 | 3.67 | 2.661 | .009 |
|  |  | Top-dog | 4.25 |  |  | 3.05 |  |  |
| Study 3 | Ethical | Underdog | 6.06 | 8.75 | .000 | 2.73 | 2.43 | .017 |
|  |  | Top-dog | 3.62 |  |  | 2.03 |  |  |
|  | Community | Underdog | 5.43 | 4.66 | .000 | 3.31 | 4.42 | .000 |
|  |  | Top-dog | 4.02 |  |  | 1.92 |  |  |
| Study 4 | Ethical | Underdog | 6.11 | 7.499 | .000 | 2.27 | 1.565 | .120 |
|  |  | Top-dog | 3.28 |  |  | 1.75 |  |  |
|  | Functional | Underdog | 5.84 | 5.636 | .000 | 3.86 | 6.004 | .000 |
|  |  | Top-dog | 3.67 |  |  | 2.54 |  |  |
|  | Community | Underdog | 5.58 | 5.638 | .000 | 4.89 | 3.901 | .000 |
|  |  | Top-dog | 3.40 |  |  | 2.85 |  |  |

# Appendix C. Summary of Within-Article Meta-Analysis


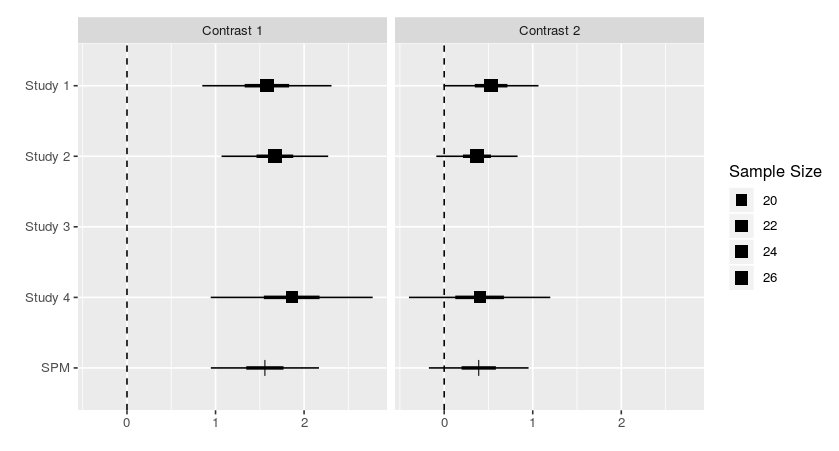

Supplement: Supplementary file 1 [file Table_1.docx]
